# Mitochondria are physiologically maintained at close to 50 °C

**DOI:** 10.1371/journal.pbio.2003992

**Published:** 2018-01-25

**Authors:** Dominique Chrétien, Paule Bénit, Hyung-Ho Ha, Susanne Keipert, Riyad El-Khoury, Young-Tae Chang, Martin Jastroch, Howard T. Jacobs, Pierre Rustin, Malgorzata Rak

**Affiliations:** 1 INSERM UMR1141, Hôpital Robert Debré, Paris, France; 2 Université Paris 7, Faculté de Médecine Denis Diderot, Paris, France; 3 College of Pharmacy, Suncheon National University, Suncheon, Republic of Korea; 4 Institute for Diabetes and Obesity, Helmholtz Centre Munich, German Research Center for Environmental Health, Neuherberg, Germany; 5 Neuromuscular Diagnostic Laboratory, Department of Pathology & Laboratory Medicine, American University of Beirut Medical Center, Beirut, Lebanon; 6 Department of Chemistry, POSTECH, Pohang, Gyeongbuk, Republic of Korea; 7 BioMediTech and Tampere University Hospital, University of Tampere, Tampere, Finland; 8 Institute of Biotechnology, University of Helsinki, Helsinki, Finland; 9 Centre National de la Recherche Scientifique, Paris, France; University College London, United Kingdom of Great Britain and Northern Ireland

## Abstract

In endothermic species, heat released as a product of metabolism ensures stable internal temperature throughout the organism, despite varying environmental conditions. Mitochondria are major actors in this thermogenic process. Part of the energy released by the oxidation of respiratory substrates drives ATP synthesis and metabolite transport, but a substantial proportion is released as heat. Using a temperature-sensitive fluorescent probe targeted to mitochondria, we measured mitochondrial temperature in situ under different physiological conditions. At a constant external temperature of 38 °C, mitochondria were more than 10 °C warmer when the respiratory chain (RC) was fully functional, both in human embryonic kidney (HEK) 293 cells and primary skin fibroblasts. This differential was abolished in cells depleted of mitochondrial DNA or treated with respiratory inhibitors but preserved or enhanced by expressing thermogenic enzymes, such as the alternative oxidase or the uncoupling protein 1. The activity of various RC enzymes was maximal at or slightly above 50 °C. In view of their potential consequences, these observations need to be further validated and explored by independent methods. Our study prompts a critical re-examination of the literature on mitochondria.

## Author summary

To ensure a stable internal temperature, endothermic species make use of the heat released during the final steps of food burning by the mitochondria present in all cells of the organism. Indeed, only a fraction of the energy released by the oxidation of respiratory substrates is used to generate ATP, while a substantial proportion is released as heat. Using a temperature-sensitive fluorescent probe targeted to mitochondria, we measured the temperature of active mitochondria in cultured intact human cells. Mitochondria were found to be more than 10 °C warmer when the respiratory chain was functional. This differential was abolished in cells depleted of mitochondrial DNA or by respiratory inhibitors but preserved or enhanced by the expression of thermogenic enzymes such as *Ciona* alternative oxidase or by uncoupling protein 1. The activity of various respiratory chain enzymes was found to be maximal near 50 °C. Note that in view of their potential consequences, the observations reported here need to be validated and explored further by independent methods.

## Introduction

As the main bioenergetically active organelles of nonphotosynthetic eukaryotes, mitochondria convert part of the free energy released by the oxidation of nutrient molecules into ATP and other useful forms of energy needed by cells. However, this energy conversion process is far from being 100% efficient, and a significant fraction of the released energy is dissipated as heat. This raises the hitherto unexplored question of the effect of this heat production on the temperature of mitochondria and other cellular components.

To address this issue, we made use of the recently developed, temperature-sensitive fluorescent probe (S1 Fig), MitoThermo Yellow (MTY) [1]. Because the fluorescence of many molecular probes is known to be sensitive to diverse factors, we investigated whether the changes in MTY fluorescence that we observed in human embryonic kidney (HEK) 293 cells could be influenced by altered membrane potential or by associated parameters, such as pH, ionic gradients, or altered mitochondrial morphology. As a major conclusion of this study, based on the fluorescence changes of MTY, we found that the rise in mitochondrial temperature due to full activation of respiration is as high as about 10 °C (*n* = 10, range 7–12 °C, compared to 38 °C, the temperature of the cell suspension medium). We also showed that respiratory chain (RC) activities measured in intact mitochondria can be increased up to threefold when assayed at the inferred mitochondrial temperature of intact cells.

## Results

**Fig 1:**
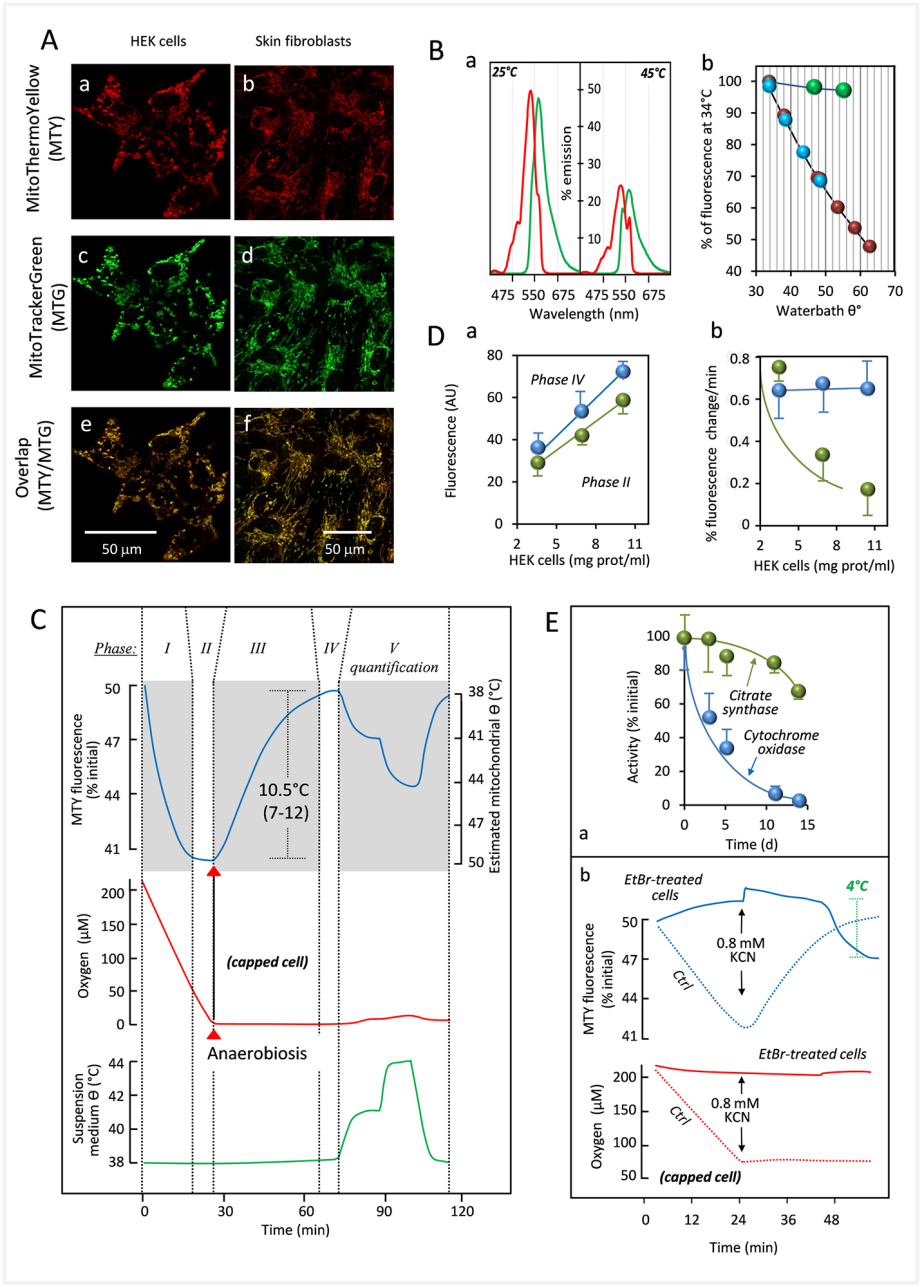
Determination of mitochondrial temperature in intact human cells. (A) The temperature-sensitive probe MTY (a, b) colocalizes with MTG (c, d), merge (e, f), in HEK293 cells and in primary skin fibroblasts, as indicated. (B) (a) Fluorescence excitation (red) and emission (green) spectra of MTY (1 mM) in 2 mL PBS at 25 and 45 °C; (b) Fluorescence response to temperature (34–64 °C) of MTY (blue and red) and rhodamine (green, also 1 mM) in 2 mL PBS. Note that the pseudo-linear decrease of MTY fluorescence corresponding to increasing temperature (blue) is essentially reversed upon cooling (red) of the solution to the initial temperature. (C) The definition of the various phases of fluorescence in MTY-preloaded HEK293 cells, as used in this study. Note that the initial value is given systematically as 50%, as set automatically by the spectrofluorometer (under these conditions; photo multiplier tension about 500 mV), allowing either increases or decreases to be recorded. Phase I: cell respiration (red trace) after cells are exposed to aerobic conditions in PBS, resulting in decreased MTY fluorescence (blue trace), as mitochondria heat up (cells were initially maintained for 10 min at 38 °C under anaerobic conditions, before being added to the cuvette). Phase II: cell respiration under aerobic conditions, in which a steady state of MTY fluorescence has been reached (maximal warming of mitochondria). Phase III: cell respiration arrested due to oxygen exhaustion—MTY fluorescence progressively increases to the starting value as mitochondria cool down. Phase IV: respiration remains stalled due to anaerobiosis; after reaching steady state, MTY fluorescence is dictated only by the water-bath temperature of 38 °C. Phase V, respiration remains stalled due to anaerobiosis, whilst temperature of the cell suspension medium (green trace) is shifted by making stepwise adjustments to water-bath temperature. Measurements were carried out in a closed quartz chamber (capped cell) except for a 0.6-mm addition hole in the handmade cap. The MTY fluorescence reached at the end of phase I was significantly different (*n* = 10; ***) from the starting value of 50%, whilst the final value in phase IV was not. (D) (a) Linear increase of fluorescence of HEK293 cells (preloaded for a minimum of 10 min, with 100 nM MTY), according to cell number (using cell protein concentration as surrogate parameter); (b) Maximal rate of decrease of MTY fluorescence (percentage, blue circles, corresponding with mitochondrial warming) is not significantly affected by cell number, whereas initial fluorescence increase in the presence of cyanide (percentage, green circles, corresponding with initial rate of mitochondrial cooling) is modulated by cell number (values at the three cell concentrations tested were significantly different from each other). (E) (a) HEK293 cells were made severely deficient for cytochrome *c* oxidase by culturing (10 days) in the presence of EtBr (1 μg/ml). Cytochrome *c* oxidase activity (blue circles) declined to a few percent of the activity measured at *t* = 0, whilst citrate synthase activity (green circles) was little changed; (b) The fluorescence of EtBr-treated HEK293 cells (10 days of EtBr treatment) preloaded with MTY (blue continuous line) does not decrease following suspension in oxygenated medium, whilst that of control HEK293 cells (blue dotted lines) follows the profile documented in Fig 1C; in contrast to control cells (red dotted line), EtBr-treated HEK293 cells also do not consume appreciable amounts of oxygen (red continuous line). Graphic drawings, means, and standard deviations are from values accessible in S1 Data. EtBr, ethidium bromide; HEK, human embryonic kidney, KCN, potassium cyanide; MTG, MitoTracker Green; MTY, MitoThermo Yellow.

We first confirmed MTY targeting to mitochondria in both HEK293 cells and primary skin fibroblasts, based on colocalization with the well-characterized dye MitoTracker Green (MTG) (Fig 1A). It was previously shown that the initial mitochondrial capture of MTY was dependent on the maintenance of a minimal membrane potential [1]. The exact sub-mitochondrial location of the probe is yet to be established, although it has been postulated to reside at the matrix side of the inner membrane [1]. MTY fluorescence from mitochondria was retained over 45 min, regardless of the presence of RC inhibitors, whilst full depolarization with an uncoupler as carbonyl cyanide *m*-chlorophenylhydrazone (*m*-Cl-CCP) led to an irreversible MTY leakage from mitochondria after only 2 min (S3 Fig). Fluorescence remained stable over 2 h in HEK293 cells, although the degree of mitochondrial MTY retention varied between cell lines, with probe aggregation observed in the cytosol in some specific lines (S3 Fig). In HEK293 cells, which were selected for further study, we observed no toxicity of MTY (100 nM in culture medium) over 2 days (S5 Fig).

We initially calibrated the response of MTY to temperature in solution. Its fluorescence at 562 nm (essentially unchanged by the pH of the solution buffer in the range 7.2–9.5) (S2 Fig), decreased in a reversible and nearly linear fashion as temperature was increased: a temperature rise from 34 to 60 °C decreased fluorescence by about 50%, whilst 82% of the response to a 3 °C shift at 38 °C was preserved at 50 °C (Fig 1Ba and 1Bb). Using a thermostated, magnetically stirred, closable 750-μl quartz-cuvette fitted with an oxygen-sensitive optode device [2], we simultaneously studied oxygen consumption (or tension) and changes in MTY fluorescence (Fig 1C). Adherent cells were loaded for a minimum of 10 min with 100 nM MTY, harvested, and washed, then kept as a concentrated pellet at 38 °C for 10 min, reaching anaerobiosis in <1 min. When cells were added to oxygen-rich buffer, they immediately started to consume oxygen (red trace; Fig 1C), accompanied by a progressive decrease of MTY fluorescence (blue trace; phase I) (Fig 1C). In the absence of any inhibitor, the fluorescence gradually reached a stable minimum (phase II). At that point, either due to a high temperature differential between the mitochondria and the surrounding cytosol (about 10 °C) and/or changes in membrane permeability leading to decreased thermal insulation, the temperature of the probe-concentrating compartment appeared to reach equilibrium. We computed the energy released as heat by the RC in this experiment as 1.05–1.35 mcal/min, based on the measured rate of oxygen consumption (11.3 ± 1.8 nmol/min/mg protein) and assuming that heat accounts for the difference between the 52.6 kcal/mol released by the full oxidation of NADH and the 21 kcal/mol conserved as ATP under a condition of maximal ATP synthesis of 3 molecules per molecule of NADH oxidized. This should be sufficient to ensure the observed thermal equilibrium (about 50 °C after 20 min) (See S1 Text). Once all the oxygen in the cuvette was exhausted (red trace), the directional shift of MTY fluorescence reversed (phase III), returning gradually almost to the starting value (phase IV). To calibrate the fluorescence signal in vivo, the temperature of the extracellular medium was increased stepwise (green trace). MTY fluorescence returned to the prior value when the medium was cooled again to 38 °C (Fig 1C, phase V). This in vivo calibration was consistent with the response of MTY fluorescence in solution up to 44 °C, although further direct calibration steps in vivo are not possible without compromising cell viability. However, we confirmed that the response of MTY fluorescence to increased temperature deviates (but only slightly) from linearity in vivo in the same manner as in aqueous solution, namely that at maximal mitochondrial warming, extrapolated as being close to 50 °C, the response to a 2 °C temperature shift is approximately 80% of that at 38 °C (S6 Fig). We therefore estimate the rise in mitochondrial temperature due to full activation of respiration as about 10 °C (*n* = 10, range 7–12 °C).

At the lowest (phase II) and highest fluorescence values (38 °C, imposed by the water bath) (phase IV), the signal was proportional to the number of added cells in a given experiment (Fig 1Da). Cell number did not affect the maximal rate of fluorescence decrease (computed from phase I). However, once anaerobic conditions had been reached, the initial rate of fluorescence increase (phase III, initial) was inversely related to the number of cells (Fig 1Db). To confirm that the observed fluorescence changes were due to mitochondrial respiration and not some other cellular process, we depleted HEK293 cells of theirmitochondrial DNA with ethidium bromide (EtBr) to a point at which cytochrome *c* oxidase activity was less than 3% of that in control cells (Fig 1Ea, S1C Fig). In EtBr-treated cells, no MTY fluorescence changes were observed upon exposure to oxygenated buffer, and cyanide treatment had no effect (Fig 1Eb).

As an additional control, we used the structurally related dye A15 (S1 Fig), which is much significantly less thermoresponsive than MTY [1]. Tested under similar conditions in HEK293 cells as MTY, no decrease of A15 fluorescence (in fact, an initial increase) was observed to accompany the activation of mitochondrial respiration by oxygenation of the medium (S8 Fig), in contrast to MTY.

**Fig 2:**
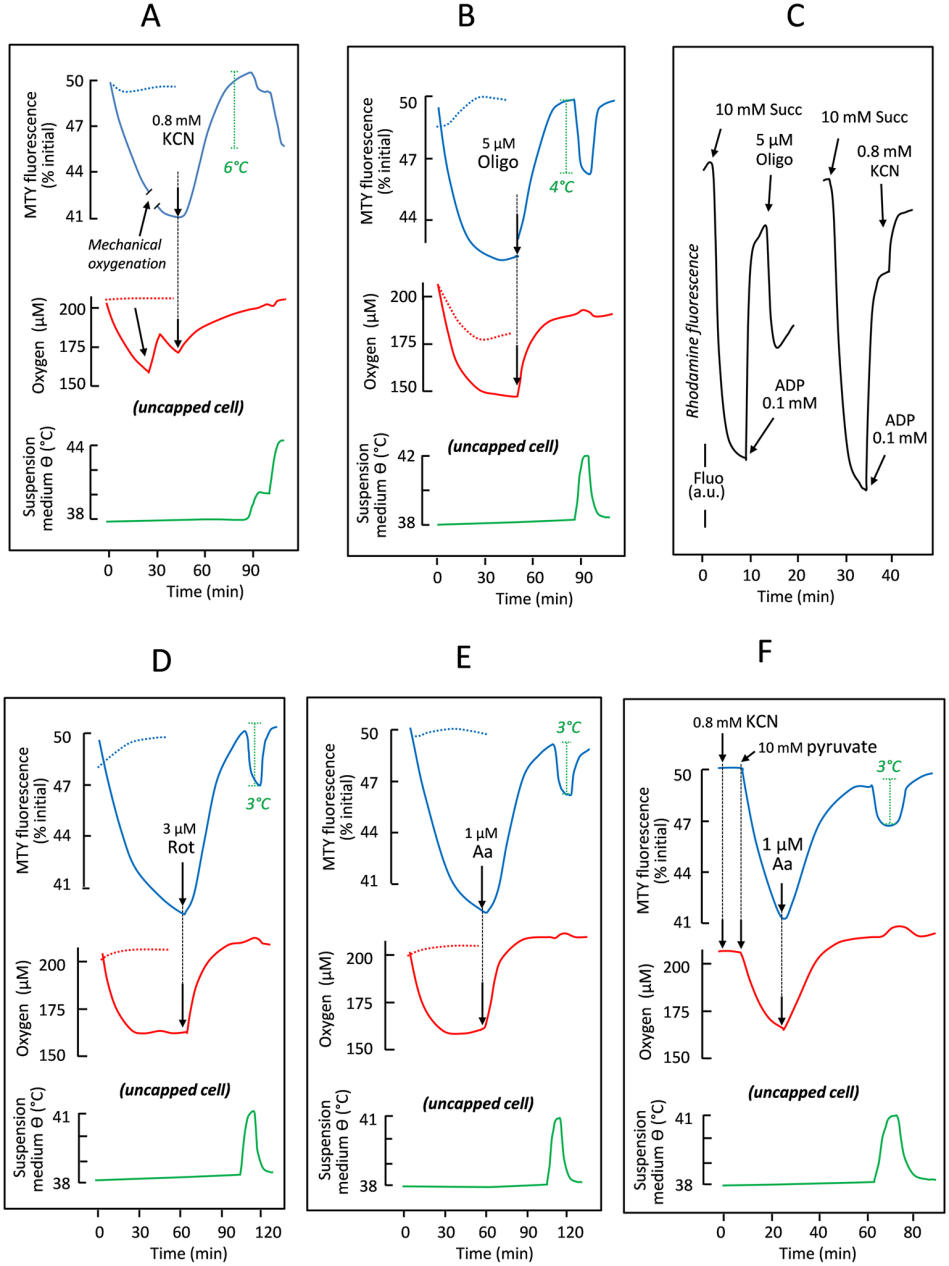
The rate of respiratory electron flow determines the temperature of mitochondria in intact HEK293 cells. (A) The effect of 0.8 mM cyanide on MTY fluorescence (blue lines) and cell respiration (red lines), when added under aerobic conditions (continuous lines), or when present from the start of the experiment (dotted lines). Changes in the temperature of the cell suspension medium (green line), imposed by water-bath adjustment, were used to calibrate the MTY fluorescence changes. (B) The effect of 5 μM oligomycin on MTY fluorescence (blue lines) and oxygen tension (affected by cell respiration balanced by medium stirring) in the uncapped quartz-cuvette (red lines), when added to freely respiring cells (continuous lines) or when present from the start of the experiment (dotted lines). (C) The effects of different inhibitors on rhodamine fluorescence, in digitonin (0.01%)-permeabilized HEK293 cells supplied with 10 mM succinate and 0.1 mM ADP, as indicated. Under state 3 conditions, 5 μM oligomycin and 0.8 mM potassium cyanide have qualitatively opposite effects on rhodamine fluorescence, used as an indicator of membrane potential (ΔΨ). The effects of 3 μM rotenone (D) and 1 μM antimycin (E) on MTY fluorescence and oxygen tension, plotted as for oligomycin in (B). (F) The effect of adding pyruvate on MTY fluorescence (blue line) and oxygen uptake (red line) by KCN-inhibited HEK293 cells. Temperature calibration (green line) of MTY fluorescence as in (A). Note that, in all experiments in which MTY fluorescence was measured prior to respiratory inhibition, the value reached at the end of phase I was in all cases significantly different (*n* ≥ 5; ***) from the starting value, whilst that in phase IV was not. Aa, antimycin A; ADP, adenosine diphosphate; a.u., arbitrary unit; HEK, human embryonic kidney; KCN, potassium cyanide; MTY, MitoThermo Yellow; Oligo, oligomycin; Rot, rotenone; Succ, succinate.

Because MTY is derived from a membrane potential–sensitive dye, the fluorescence of which is essentially unaffected by temperature (Fig 1Bb), we investigated whether the observed changes in MTY fluorescence could be influenced by altered membrane potential or by associated parameters. We took advantage of the fact that cyanide or oligomycin exert opposite effects on membrane potential (Fig 2C, S1 Fig) as well as on pH [3] and on the condensed versus orthodox state of the mitochondrial matrix [4,5], and we compared the response of MTY fluorescence to these inhibitors (Fig 2A and 2B). To avoid the possibly confounding effect of anaerobiosis, the quartz cuvette was kept uncapped in this experiment, with the oxygen tension rather than the rates of oxygen uptake being recorded (red traces). Once MTY fluorescence was stabilized (assumed to represent maximal mitochondrial heating) and the suspension buffer re-oxygenated, cyanide was added, causing a progressive increase in MTY fluorescence to the starting value (Fig 2A). Note that, when cyanide was initially present, fluorescence changes and oxygen uptake were both abolished (Fig 2A, dotted lines). Adding oligomycin in lieu of cyanide also decreased oxygen consumption and, as observed with cyanide, brought about a similar increase in MTY fluorescence (Fig 2B). If added first, oligomycin progressively decreased oxygen uptake, simultaneously abolishing the decrease in MTY fluorescence (Fig 2B, dotted lines). Taken together, these experiments imply that electron flow through the RC rather than membrane potential or any related factor influences MTY fluorescence, which can thus be interpreted as a surrogate for mitochondrial temperature. Examining the respective kinetics of membrane potential changes (tens of seconds) and MTY fluorescence changes (tens of minutes) supports this conclusion.

A similar effect was observed with two other respiratory inhibitors (S1 Fig), affecting either RC complex I (CI; rotenone, Fig 2D) or III (CIII; antimycin, Fig 2E). Despite their different effects on the redox state of the various RC electron carriers, these inhibitors blocked oxygen uptake and again triggered an increase in MTY fluorescence. Importantly, these two inhibitors (and oligomycin) have been shown to trigger increased production of superoxide by the RC [6], but their effects on MTY fluorescence are similarly determined by oxygen consumption as for cyanide inhibition, which decreases superoxide production at complex III. This excluded any interference from superoxide in the observed MTY fluorescence changes. Taking advantage of cyanide removal from cytochrome *c* oxidase to form cyanohydrin in the presence of α-ketoacids under aerobiosis [7], we confirmed that the blockade of the RC did not result in MTY leakage from the mitochondria because pyruvate addition resulted in the resumption of oxygen uptake and a renewed decrease in MTY fluorescence, both of which were inhibited by a further addition of antimycin (Fig 2F). Leakage of the probe from mitochondria of other cell lines was reflected in a decreased ability of cyanide to restore MTY fluorescence to its initial value (S4 Fig).

**Fig 3:**
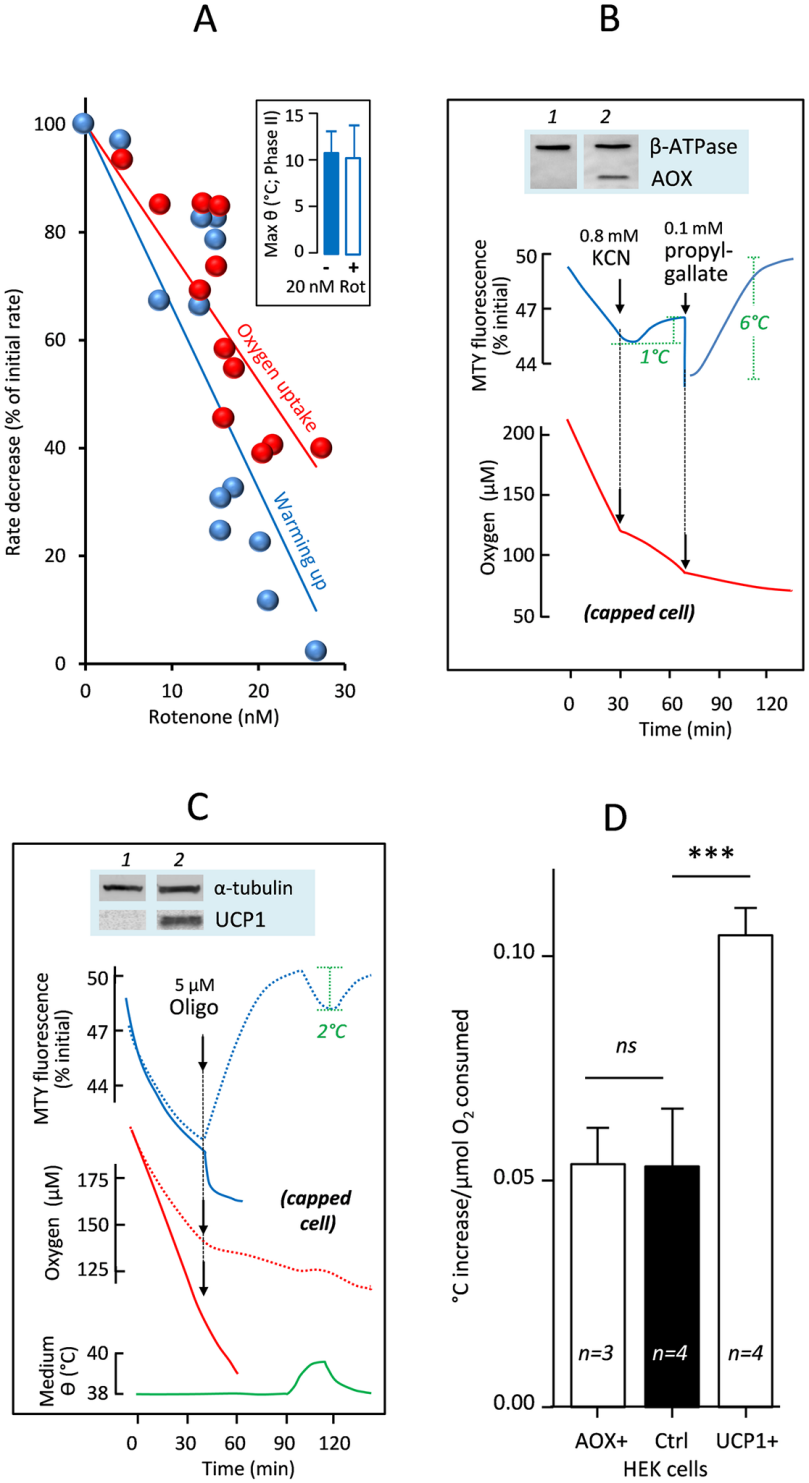
Effects on mitochondrial temperature of respiratory inhibitors, uncouplers, and expression of heterologous mitochondrial proteins. (A) Effect of variable rotenone addition to control HEK293 cells on the rates of oxygen uptake and fluorescence decrease of MTY. Rotenone was added at *t* = 4 min; rates calculated from 4–7 min and expressed as a percent of initial rate. Inset: maximal warming of HEK293 cell mitochondria in the absence or presence of 20 μM rotenone. (B, C) Changes in MTY fluorescence (blue lines), cell respiration (B, C) (red lines), and temperature of cell suspension medium (green line), with additions of inhibitors as shown (potassium cyanide, n-propyl gallate, and/or oligomycin), alongside western blots confirming expression of the indicated genes: *Ciona intestinalis* AOX (B), UCP1 (C), alongside loading controls, as indicated. Traces for control cells are shown by dotted lines. The abrupt change in fluorescence upon addition of n-propyl gallate is due to the absorbance of the chemical itself. Note that in all experiments in which MTY fluorescence was measured, the value reached at the end of phase I was in all cases significantly different from the starting value, whilst that in phase IV was not (*n* = 4; ***). (D) Computed from experiments using HEK293 cells endowed with AOX (AOX+), UCP1 (UCP1+) or control cells, initial increases of temperature (°C) per μmol oxygen consumed were compared and statistically analyzed by one-way ANOVA with Bonferroni’s multiple comparison test (*n* = 3–4; means ± SD). Graphic drawings, means, and standard deviations are from values accessible in S1 Data. AOX, alternative oxidase; Ctrl, control; HEK, human embryonic kidney; KCN, potassium cyanide; MTY, MitoThermo Yellow; ns, nonsignificant; Oligo, oligomycin; Rot, rotenone; UCP1, uncoupling protein 1.

Note that changes in MTY fluorescence cannot be attributed to altered mitochondrial morphology, since 45 min of MTY treatment had no detectable effect on the mitochondrial network (S3 Fig, panels i,j), nor did any of the inhibitors used in the above experiments (S3 Fig, panels a–f). Similarly, MTY fluorescence changes appear to be independent of oxygen tension as (1) a similar loss of fluorescence was observed, regardless of whether oxygen tension was maintained at a constant level (capped or uncapped cell; e.g., Figs 1C versus 2A or 2B); (2) MTY fluorescence was not modified by mechanical re-oxygenation of the medium (Fig 2A); and (3) using cells expressing the cyanide-insensitive nonproton motive alternative oxidase (AOX) from *C*. *intestinalis*, MTY fluorescence remained stable after the addition of cyanide, even though oxygen continued to be consumed (Fig 3B after cyanide addition). Importantly, the fluorescence of the endoplasmic reticulum (ER)-targeted probe ER thermo yellow [8] in both HEK293 cells and skin fibroblasts was essentially unaffected by the activity of the mitochondria when modulated by cyanide, pyruvate, or antimycin (S7 Fig), although it was sensitive to externally imposed (water-bath) temperature changes.

We next studied MTY probe behavior in HEK293 cells, in which CI was inhibited by the addition of varying amounts of rotenone (Fig 3A). The rate of change of MTY fluorescence was proportional to the residual respiratory electron flux whilst the maximal temperature, as implied by MTY fluorescence at equilibrium (phase II), was essentially unchanged (Fig 3A, inset). We next tested the effect of expressing AOX (Fig 3B, S1 Fig), the activity of which is unmasked in the presence of cyanide [8]. Before cyanide addition, the decrease in MTY fluorescence in AOX-expressing cells was similar to control cells, consistent with previous inferences that the enzyme does not significantly participate in uninhibited cell respiration [9]. However, upon cyanide addition, AOX-endowed cells maintained low MTY fluorescence (Fig 3B, blue trace), whilst the rate of oxygen consumption decreased by more than 50% (red trace). The implied increase in the ratio of heat generated to respiration is consistent with the predicted thermogenic properties of AOX. Subsequent addition of 0.1 mM propylgallate, which inhibits AOX, almost completely abolished the residual respiration and brought MTY fluorescence back to its starting value (corresponding to 38 °C).

So as to circumvent the fact that we were not able to use chemical uncouplers with this probe [1], we used HEK293 cells engineered to express the uncoupling protein 1 (UCP1) (Fig 3C, S1 Fig), which diminishes membrane potential and shifts the balance of respiratory energy conversion from ATP synthesis towards heat production. As expected, UCP1 conferred an increased rate of respiration, which was only partially inhibited by oligomycin (red trace) and accompanied by an even greater drop in MTY fluorescence. Based on the internal calibration at the end of the experiment, this is equivalent to a temperature of about 12 °C above the cellular environment. HEK293 cells expressing UCP1 also exhibited a faster rate of MTY fluorescence decrease compared with control HEK293 cells, about twofold during the first 5 min. Importantly, the expression of UCP1 did not slow down the rate of MTY fluorescence decrease as would be predicted if this decrease were dependent on membrane potential or pH gradient (Fig 3D).

**Fig 4:**
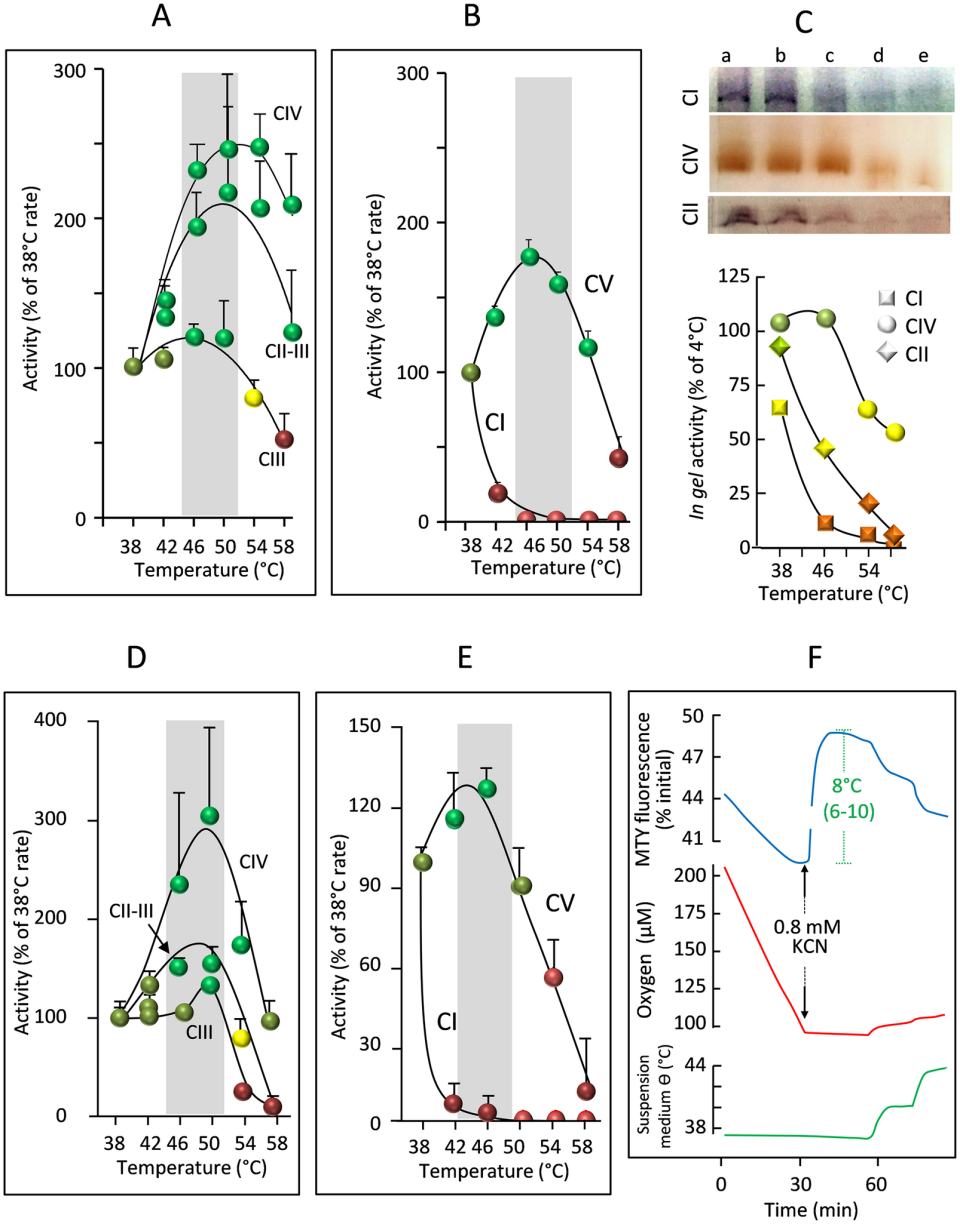
Effects of assay medium temperature on RC activities. (A, D) Temperature profile of cytochrome *c* oxidase (CIV), malonate-sensitive succinate:cytochrome *c* reductase (CII+CIII), and antimycin-sensitive decylubiquinol:cytochrome *c* reductase (CIII) activity in (A) HEK293 cells and (D) primary skin fibroblasts, after two freeze–thaw cycles, and (B, E) oligomycin-sensitive ATPase (CV) and rotenone-sensitive NADH:decylubiquinone reductase (CI) activity, after disruption of inner mitochondrial membrane in frozen (B) HEK293 cells and (E) primary skin fibroblasts by osmolysis with water [10]. Colors denote optimal (green), minimal (red), and degrees of intermediate (pale greens, yellow) activity. Grey bars indicate optimal temperature range. (C) clear native electrophoresis (CNE) in-gel activities of CI, CIV, and CII extracted from mitochondria previously incubated for 10 min in water at (a) 4 °C, (b) 37 °C, (c) 42 °C, (d) 46 °C, and (e) 55 °C, also plotted graphically (lower panel). (F) Changes in MTY fluorescence (blue), cell respiration (red), and temperature of cell suspension medium (green) for primary skin fibroblasts, as denoted in Fig 2A, with the addition of KCN, as shown. Graphic drawings, means, and standard deviations are from values accessible in S1 Data. HEK, human embryonic kidney; KCN, potassium cyanide; MTY, MitoThermo Yellow; RC, respiratory chain.

The surprisingly high inferred mitochondrial temperatures prompted us to test the dependence on assay medium temperature of RC enzyme activities measured under V_max_ conditions in crude extracts, in which mitochondrial membrane integrity is maintained (Fig 4A and 4B). Antimycin-sensitive CIII, malonate-sensitive succinate cytochrome *c* reductase (CII+CIII), and cyanide-sensitive cytochrome *c* oxidase (CIV) activities all showed temperature optima at or slightly above 50 °C, whilst these activities tended gradually to decrease as the temperatures were raised further (Fig 4A). This was not so for those enzymes whose activities can be measured in vitro only after osmotic disruption of both outer and inner mitochondrial membranes (Fig 4B). Oligomycin-sensitive ATPase (CV) activity was optimal around 46 °C, whereas rotenone-sensitive NADH quinone-reductase (CI) activity declined sharply at temperatures above 38 °C. After disruptive treatment, the activities of CIV and CII of mitochondria, as revealed by native electrophoresis and in-gel activity (IGA) (Fig 4C), were also impaired at high temperature. Taken together, these findings strongly suggest a vital role for the integrity of the inner mitochondrial membrane structure in the stabilization of the RC complexes at high temperature. We next analyzed the temperature profile of RC activity of primary skin fibroblasts. For CII+CIII, CIII, and CIV (Fig 4D) as well as CV (Fig 4E), similar temperature optima were observed as in HEK293 cells, whilst MTY fluorescence (Fig 4F) also indicated mitochondria being maintained at least 6–10 °C above environmental temperature.

## Discussion

Our data raise many questions: have we excluded all possible artifacts and confounding factors influencing MTY fluorescence? Is the inference that mitochondria operate at temperatures of 48 °C or more credible, in light of theoretical considerations? Are the findings consistent with those reported elsewhere? What are their implications for the structure, function, and pathology of mitochondria?

For several years, an intense debate has addressed the possibility of maintaining temperature gradients in isolated cells, considering the small volumes involved [11,12,13,14,15]. Largely based on theoretical considerations, it has been suggested that factors other than temperature could account for the large fluorescence changes observed using thermosensitive probes [14]. For mitochondria, these potential factors include membrane potential changes (and related changes in pH, ionic gradients, and matrix morphology), altered mitochondrial superoxide production, varying oxygen concentration, changes in probe conformation (especially for fluorescent proteins), or probe leakage from mitochondria. Using indirect methods, we were able to exclude most of these factors from significantly influencing intracellular MTY fluorescence under our experimental conditions. Previous studies of MTY in aqueous solution already excluded changes in pH, viscosity, metal ions, and oxygen species from affecting its fluorescence [1], although it remains formally possible that this nonresponsiveness is modified under cellular conditions. More direct measurements, as successfully implemented for the ER-targeted probes ER thermo yellow [8] and its derivative ERthermAC [17] in regard to Ca^2+^ levels and pH, are precluded by the fact that MTY requires a minimal membrane potential to be taken up and retained inside mitochondria, preventing meaningful studies from being carried out using fixed cells. Despite this caveat, we observed a consistent and robust correlation between MTY fluorescence and temperature, unaffected by or unrelated to implied changes in any other parameter. However, these observations will need to be validated and explored further by independent methods.

Uncertainties regarding micro- and nanoscale physical parameters [15] render purely theoretical considerations questionable when considering the complex and dynamic structure of mitochondria. Most models have treated mitochondria as isolated, undifferentiated balls, floating in an aqueous medium, the cytosol. In considering temperature diffusion, the situation is often depicted as a thin membrane separating two compartments, whilst the MTY-containing space should be more accurately depicted as a narrow zone sandwiched between heat-producing membranes. Structural oversimplification may underestimate significant temperature differences between mitochondria and the cytosol. Furthermore, mitochondria in vivo typically form an interconnected filamentous network, variously packed according to cell type. Assuming MTY to be localized to the inner face of the inner membrane or the adjacent pockets of matrix, the heated compartments would be juxtaposed to each other rather than to the colder cytosol. Moreover, compaction of the cytosol in domains rich in mitochondria, as observed in HEK293 cells, would also limit heat conduction out into the rest of the cell.

The molecular heterogeneity of the various submitochondrial compartments must also be considered. The inner membrane is rich in proteins (protein-to-lipid ratio is 80:20, compared to 50:50 for the outer membrane), including those that are sources of heat, and has a distinct lipid composition, including cardiolipins. The intermembrane space, the phospholipid-rich outer membrane, the cytosol, plus the plasma membrane potentially represent additional layers of insulation from the cell suspension medium, with many relevant parameters unknown, including heat conductance and geometry of the various compartments. Measuring how the lipid composition of the mitochondrial membranes influences the thermal stability of the RC complexes and the temperature-responsiveness of other mitochondrial properties, such as swelling and the permeability transition, will shed light on these issues. It will also be interesting to compare the content of cardiolipins and other lipids in mitochondrial membranes from homeotherms and from poikilotherms living at much colder environmental temperatures.

A 6–9 °C temperature shift between mitochondria and the surrounding cytosol, induced by the addition of an uncoupler (10 μM carbonyl cyanide-4-(trifluoromethoxy)phenylhydrazone) was recently reported in HeLa cells [16] using a genetically encoded, green fluorescent protein-derived ratiometric fluorescent probe. Although carried out under quite different conditions (confocal microscopy of a single cell) and without determining mitochondrial activity under these conditions, the data are consistent with our own findings. In addition, the investigation of the temperature of the ER in activated brown adipocytes using ERthermAC as a probe [17] concluded that it was able to reach temperatures over 15 °C hotter than the surrounding medium. Noting that the ER in these cells is in close juxtaposition to mitochondria, which are the source of heat, this would be consistent with brown fat mitochondria being maintained at temperatures even exceeding those we inferred in the present study.

The physical, chemical, and electrical properties of the inner mitochondrial membrane and of mitochondria in general should be carefully considered in light of our findings. Most previous literature reflects experiments conducted far from our inferred physiological temperature, implying a need to expand this voluminous body of work to take account of it.

Traditional views of the lipid component of the respiratory membrane as a lake in which the RC complexes are freely diffusing, or as a sealant occupying the space between tightly packed proteins [17], may need to be revised to consider it as a glue that maintains the integrity of the respiratory complexes.

Organisms survive across a wide range of temperatures, ranging from below 0 °C to above 100 °C, and their enzymes and membranes have adapted to function accordingly [20,21], in addition to other adaptations that insulate them from the external environment. For example, the NADP-dependent isocitrate dehydrogenase of thermophilic bacteria functions efficiently above 70 °C [18]. Thus, there is no theoretical reason why enzymes of mammalian mitochondria could not tolerate the temperatures implied by our findings. Currently, rather little information is available on the thermal stability, temperature optima, and thermosensitive properties of mitochondrial enzymes. One well-documented case indicates that the thermal stability of purified NADP-dependent isocitrate dehydrogenase from nonthermophiles [19] is dependent on its substrate, isocitrate, and on its cofactor, magnesium, leading to full protection of activity at 60 °C for 2 h, conditions that otherwise readily inactivate it. It will obviously be instructive to evaluate the relevant parameters of mammalian mitochondrial enzymes under conditions as close as possible to those encountered physiologically and to try to interpret such data in light of the actual range of temperatures of different submitochondrial and subcellular compartments, once temperature-responsive probes for these locations become available.

The effects of respiratory dysfunction may need to be reconsidered, taking account of temperature changes that could impact membrane fluidity, electrical conductance, and transport. The organization of RC supercomplexes [20,21] should be re-examined using methods less disruptive than CNE The subcellular distribution of mitochondria (e.g., perinuclear, or synaptic) has previously been considered to reflect ATP demand, but mitochondria should also be regarded as a source of heat, potentially relevant in specific cellular or physiological contexts, not just in specifically thermogenic tissues like brown fat. Furthermore, temperature differences should be considered as an additional possible dimension to the intracellular functional heterogeneity of mitochondria.

Fully resolving how heat is conducted inside the cell will require the development of fluorescent or fluorescently tagged temperature-responsive probes tightly targeted to specific subcellular and submitochondrial locations, to report on their temperature and how it changes under specific physiological conditions.

## Materials and methods

### Cell culture

Human cells derived from embryonic kidney, HEK293, hepatoma tissue culture, HTC-116, and large-cell lung cancer, NCI-H460 (American Type Culture Collection, Manassas, VA), as well as primary skin fibroblasts derived from healthy individuals and HEK293 cells expressing *C*. *intestinalis* AOX [22] or UCP1 [23,24] were cultured in DMEM medium containing 4.5 g/L glucose and 2 mM glutamine (glutamax; Gibco Thermo Fisher Scientific, MA), 10% fetal calf serum, 200 μM uridine, 2 mM pyruvate, 100 U/mL each penicillin and streptomycin. The trypan blue exclusion test was used to determine the number of viable and dead HEK293 cells [25].

### Immunoblot analyses and in-gel enzyme activity assays

For western blot analysis, mitochondrial proteins (50 μg) were separated by SDS–PAGE on a 12% polyacrylamide gel, transferred to a nitrocellulose membrane, and probed overnight at 4 °C with antibodies against the protein of interest, AOX 1:10,000 [26], UCP1 1:10,000 [27]. Membranes were then washed in TBST and incubated with mouse or rabbit peroxidase-conjugated secondary antibodies for 2 h at room temperature. The antibody complexes were visualized with the Western Lightning Ultra Chemiluminescent substrate kit (Perkin Elmer). For the analysis of RC complexes, mitochondrial proteins (100 μg) were extracted with 6% digitonin and separated by hrCN-PAGE on a 3.5%–12% polyacrylamide gel. Gels were stained by IGA assay detecting CI, CII, and CIV activity, as described [28].

### Staining procedures and life cell imaging

Cells were seeded on glass coverslips and grown inside wells of a 12 well plate for 48 h in standard growth media at 37 °C, 5% CO2. The culture medium was replaced with prewarmed medium containing fluorescent dyes, namely 100 nM MTG (Invitrogen M7514) and 100 nM MTY [1] or 100 nM ER thermo yellow [29]. After 10 min, the staining medium was replaced with fresh prewarmed medium or PBS and cells were observed immediately by Leica TCS SP8 confocal laser microscopy.

### Assay of mitochondrial RC activity

The measurement of RC activities was carried out using a Cary 50 spectrophotometer (Varian Australia, Victoria, Australia), as described in [30]. Protein was estimated using the Bradford assay.

### Simultaneous spectrofluorometric, temperature, oxygen uptake assay

Detached subconfluent HEK293, NCI-H460, or HTC-116 cells (25 cm^2^ flask) or trypsinized subconfluent skin fibroblasts (75 cm^2^ flask) were treated for a minimum of 10 min with 100 nM MTY (or 100 nM compound A15 [1]) in 10 mL DMEM and recovered by centrifugation at 1,500 *g*_*max*_ for 5 min. The pellet was washed once in 1 mL PBS, then maintained as a concentrated pellet. After anaerobiosis (checked by inserting an optic fiber equipped with an oxygen-sensitive fluorescent terminal sensor [Optode device; FireSting O_2_, Bionef, Paris, France]) was established (10 min incubation of the pellet at 38 °C), cells (1 mg prot) were added to 750 μl PBS thermostatically maintained at 38 °C. The fluorescence (excitation 542 nm, emission 562 nm for MTY; excitation 559 nm, emission 581 nm for ER thermo yellow; excitation 500 nm, emission 520 nm for A15), the temperature of the medium in the cuvette, and the respiration of the intact cell suspension were simultaneously measured in a magnetically stirred, 38 °C-thermostated 1-mL-quartz cell using a Xenius XC spectrofluorometer (SAFAS, Monaco). Oxygen uptake was measured with an optode device fitted to a handmade cap, ensuring either closure of the quartz cell yet allowing micro-injections (hole with 0.6-mm diameter), or leaving the quartz cell open to allow for constant oxygen replenishment. Alternatively, untreated HEK293 cells (250 μg protein) were added to 750 μL of buffer consisting of 0.25 M sucrose, 15 mM KCl, 30 mM KH_2_PO_4_, 5 mM MgCl_2_, 1 mM EGTA, pH 7.4, followed by the addition of rhodamine to 100 nM and digitonin to 0.01% w/v. The permeabilized cells were successively given a mitochondrial substrate (10 mM succinate) and ADP (0.1 mM) to ensure state 3 (phosphorylating) conditions, under which either 5 μM oligomycin or 0.8 mM cyanide was added.

### Statistics

Data are presented as mean ±SD. Statistical significance was calculated by standard unpaired one-way ANOVA with Bonferroni posttest correction; a *p* value <0.05 was considered statistically significant (GraphPad Prism).

## Supporting information

S1 Fig The structures of MTY and the related probe A15, with a series of schematized views of the various conditions used for testing mitochondrial heating in situ in cells. (A) The structures of the rosamine-derived MTY and A15 probes. (B) The concurrent synthesis of ATP and heat generation by the respiratory chain. (C) The defective respiratory chain of EtBr-treated cells. (D) The sites of action of the several inhibitors used in this study. The respiratory chain of AOX- (E) or UCP1-expressing (F) HEK cells. I, II, III, IV, V, the various complexes of the respiratory chain; Aa, antimycin A; AOX, alternative oxidase; EtBr, ethidium bromide; Fum, fumarate; HEK, human embryonic kidney; MTY, MitoThermo Yellow; Oligo, oligomycin; Q, ubiquinone 10 (coenzyme Q); Rot, rotenone; Succ, succinate; UCP1, uncoupling protein 1.
(PDF)

S2 Fig The effect of temperature on pH of PBS buffer, and of pH on MTY fluorescence in solution. (A) Temperature did not detectably affect the pH value of PBS in the range of temperature studied (36–50 °C). (B) Change of PBS pH from 6.8 to 9.5 has only a minimal effect (<3%) on the fluorescence of MTY (1 mM). Approximately a 1% change in fluorescence was recorded as pH was raised from 7.2 to 8.0 (physiological range). Of note, a pH value of 7.8 for the mitochondrial matrix has been determined in human ECV304 (ECACC 92091712) cells [31]. Graphic drawings, means, and standard deviations are from values accessible in S2 Data. MTY, MitoThermo Yellow.
(PDF)

S3 Fig Treatment with carbonyl cyanide m-chlorophenylhydrazone (*m*-Cl-CCP; 1 μM) rapidly causes leakage of the MTY probe from the mitochondria of intact cells, at variance with respiratory chain inhibitors, KCN, rotenone, or oligomycin. (a, c, e, g, i) At *t*_*0*_, before the addition of the drugs, the mitochondrial network of plated primary skin fibroblasts is clearly visible when stained with the MTY probe. After 45 min, no significant change in staining was observed when no inhibitor was present (j) or when KCN (1 mM) (b), rotenone (0.3 μM) (d), or oligomycin (0.5 μM) (f) were added. In contrast, the addition of *m-*Cl-CCP (1 μM) (h) rapidly causes leakage of the probe from the mitochondria, and after just 2 min, MTY appears mostly as a diffuse staining of the cytosol. Noticeably, in the presence of *m*-Cl-CCP (or valinomycin) treatment, anaerobiosis was unable to trigger any changes in MTY fluorescence monitored, as in Fig 1C. m-Cl-CCP, carbonyl cyanide m-chlorophenylhydrazone; MTY, MitoThermo Yellow.
(PDF)

S4 Fig MTY probe retention in different cell lines. (A) Initially, MTY fluorescence is mostly localized to mitochondria in HEK293 (a), large cell lung cancer-derived cells (NCI-H460) (b), and colorectal carcinoma-derived cell line (HTC-116) (c) cell lines, as shown by the overlapping staining of MTY (shown in red) and MitoTracker green (overlapping shown in yellow). After 2 h, a significant amount of the probe is excluded from mitochondria in NCI cells (e), resulting in many cells in which green- and red-colored fluorescence is no longer fully colocalized. Notably, red (MTY) fluorescence is observed in small cytosolic granules (h). A similar but more pronounced phenomenon is observed in HTC cells, in which large granules can be observed (f, i, j). (B) Quantification of MTY-stained (red) granules (a) and clustered granules (b) in HEK, NCI, and HTC cells. (C) MTY-fluorescence changes (as in Fig 2A) in HEK, NCI, and HTC cells upon shifting from anaerobic to aerobic conditions and the effect of a subsequent addition of cyanide. Note that, while cyanide restores the initial fluorescence value in HEK cells, it does not do so in NCI and even less in HTC cells. Taken together, these experiments indicated that, depending on cell type, MTY can either be retained for at least 2 h in mitochondria (HEK cells) or excluded with variable kinetics as cytosolic granules (NCI, HTC cells), with an irreversible loss of MTY fluorescence, as measured in the spectrofluorometer quartz cuvette (C). Graphic drawings, means, and standard deviations are from values accessible in S2 Data. HEK, human embryonic kidney; MTY, MitoThermo Yellow.
(PDF)

S5 Fig MTY has no detectable effect on HEK293 cell viability. Cell counts were similar for living or dead HEK cells in the absence (−) or presence (+) of 100 nM MTY at 48 h. As a result, cell viability does not appear to be affected by MTY at this concentration. Graphic drawings, means, and standard deviations are from values accessible in S2 Data. HEK, human embryonic kidney; MTY, MitoThermo Yellow.
(PDF)

S6 Fig Fluorescence response of mitochondrially localized MTY to temperature change is affected by its actual temperature (50 °C or 38 °C). As shown in Fig 1Bb, the fluorescence response of MTY probe to temperature change tends to be decreased at high temperature compared to the response at 38 °C. At 50 °C (maximal decrease of MTY fluorescence), similarly to the probe in solution, a 2 °C shift results in 80% of the response observed at 38 °C. At such a high temperature, a 1% fluorescence change represents 1.4 °C, compared to 1.12 °C at 38 °C. HEK, human embryonic kidney; MTY, MitoThermo Yellow.
(PDF)

S7 Fig Modulating respiratory chain activity does not change the fluorescence of an endoplasmic reticulum-targeted temperature probe (ERTY). (A) ERTY (red) and MitoTracker green (green) fluorescence did not overlap (bottom) in HEK cells. (B) Tested in the same way as MTY in the spectrofluorometer (Fig 1), the fluorescence of ERTY within HEK293 cells (brown line) was unaffected by the activity of the mitochondria when modulated by cyanide, pyruvate, or antimycin, chemicals that influenced oxygen uptake (red line). Notably, the initial fluorescence decrease of ERTY was similar in the absence or presence of cyanide (not shown). (C) ERTY (red) and MitoTracker green (green) fluorescence also did not overlap in skin fibroblasts (C), in contrast to MTY and MitoTracker green, the staining of which was perfectly overlapping in the same cells (Fig 1Af). ERTY, ER Thermo Yellow; HEK, human embryonic kidney; MTY, MitoThermo Yellow.
(PDF)

S8 Fig A15 fluorescence changes in HEK293 cells following the activation of mitochondrial respiration. The temperature response of the MTY-related dye A15 (structure shown in S1 Fig) inside cells was previously shown to be only approximately 10% that of the temperature response of MTY itself [1]. Thus, it provides a useful control as to whether the drop in fluorescence of MTY observed in response to the activation of mitochondrial respiration is due to the previously demonstrated temperature-responsiveness of MTY, or to some other property of this family of dyes. As shown here, when cells were loaded with A15 instead of MTY and respiration was activated by oxygenation of the medium, the fluorescence changes were very different from those observed with MTY itself. Whereas MTY fluorescence showed a reversible decrease in response to respiratory activation (Fig 1C), A15 fluorescence initially showed an increase, subsequently stabilizing and drifting slightly downwards as oxygen consumption declined. Of note, the photomultiplier tension determined by the fluorimeter to result in an initial 50% fluorescent signal (condition of Fig 1C) was 650 mV with A15 (about 500 mM for MTY). This difference renders hazardous a quantitative comparison between the recorded signals with the two probes. Nevertheless, the opposite behavior of A15 is consistent with the fluorescence changes of MTY, reflecting the specific properties of the latter as a temperature sensor. HEK, human embryonic kidney; MTY, MitoThermo Yellow.
(PDF)

S1 Data The measured values that were used as underlying data to calculate means and standard deviation in Figs 1Bb, 1Ea, 1Da, 1Db and 3A and inset, Figs 3D, 4A, 4B, 4C, 4D and 4E. (XLSX)

S2 Data The measured values that were used as underlying data to calculate means and standard deviation in the S2A, S2B, S4Ba, S4Bb and S5 Figs. (XLSX)

S1 Text A quantitative estimation of the heat releases by a functional respiratory chain based on the substrate consumption per minute and milligram protein. (DOCX)

## Abbreviations

AOX: alternative oxidase
CI: complex I (NADH dehydrogenase)
CII: complex II (succinate dehydrogenase)
CIII: complex III
CIV: complex IV (cytochrome c oxidase)
CNE: clear native electrophoresis
CV: complex V (ATPase)
ER: endoplasmic reticulum
EtBr: ethidium bromide
HEK: human embryonic kidney
IGA: in-gel activity
*m*-Cl-CCP: carbonyl cyanide *m*-chlorophenylhydrazone
MTG: MitoTracker Green
MTY: MitoThermo Yellow
RC: respiratory chain
UCP1: uncoupling protein 1
