# Supplementary material for: Mitochondria are physiologically maintained at close to 50 °C
Source: PLoS Biol. 2018 Jan 25;16(1):e2003992. doi: 10.1371/journal.pbio.2003992 (PMC5784887; doi:10.1371/journal.pbio.2003992)
Supplement: S1 Fig — (A) The structures of the rosamine-derived MTY and A15 probes. (B) The concurrent synthesis of ATP and heat generation by the respiratory chain. (C) The defective respiratory chain of EtBr-treated cells. (D) The sites of action of the several inhibitors used in this study. The respiratory chain of AOX- (E) or UCP1-expressing (F) HEK cells. I, II, III, IV, V, the various complexes of the respiratory chain; Aa, antimycin A; AOX, alternative oxidase; EtBr, ethidium bromide; Fum, fumarate; HEK, human embryonic kidney; MTY, MitoThermo Yellow; Oligo, oligomycin; Q, ubiquinone 10 (coenzyme Q); Rot, rotenone; Succ, succinate; UCP1, uncoupling protein 1. (PDF) [file pbio.2003992.s001.pdf]

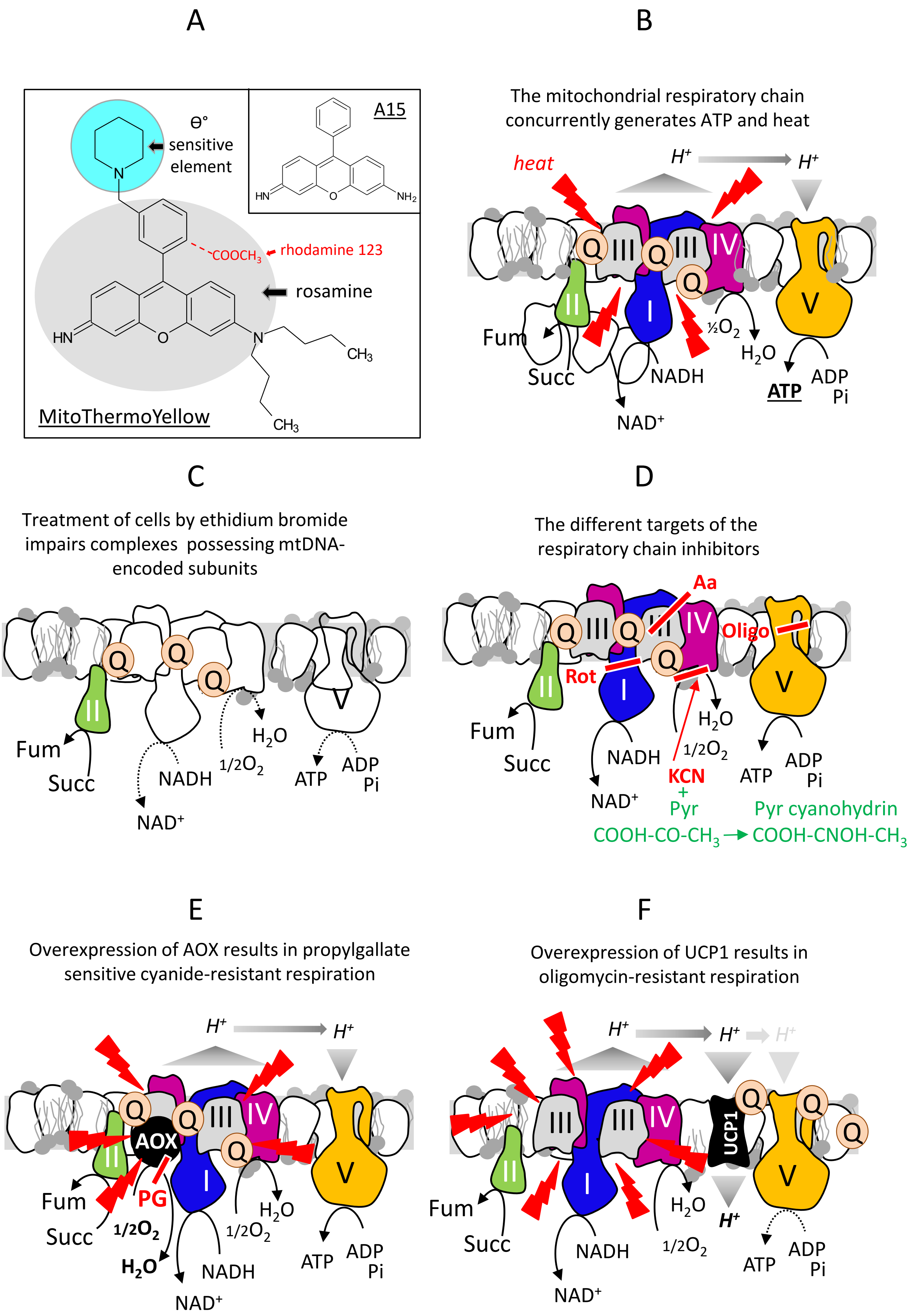
A
B
θ°
sensitive
element
A15
HN
O
NH2
COOCH3
rhodamine 123
rosamine
HN
O
N
CH3
CH3
MitoThermoYellow
The mitochondrial respiratory chain
concurrently generates ATP and heat
heat
H+
H+
Q
III
Q
III
IV
Q
II
I
V
Fum
Succ
NADH
NAD+
½O2
H2O
ATP
ADP
Pi
C
D
Treatment of cells by ethidium bromide
impairs complexes possessing mtDNA-
encoded subunits
Q
Q
Q
II
V
Fum
Succ
NADH
NAD+
H2O
1/2O2
ATP
ADP
Pi
The different targets of the
respiratory chain inhibitors
Aa
Q
III
Q
III
IV
Oligo
Q
Rot
II
I
V
Fum
Succ
NADH
NAD+
H2O
1/2O2
KCN
+
Pyr
ATP
ADP
Pi
Pyr cyanohydrin
COOH-CO-CH3
COOH-CNOH-CH3
E
F
Overexpression of AOX results in propylgallate
sensitive cyanide-resistant respiration
H+
H+
Q
Q
III
IV
AOX
Q
II
I
V
PG
Fum
Succ
1/2O2
H2O
1/2O2
H2O
NADH
NAD+
ATP
ADP
Pi
Overexpression of UCP1 results in
oligomycin-resistant respiration
H+
H+
H+
III
III
IV
UCP1
Q
Q
Q
II
I
V
Fum
Succ
NADH
NAD+
H2O
1/2O2
H+
ATP
ADP
Pi
