# Supplementary figures and images for: Mitochondria are physiologically maintained at close to 50 °C
Source: PLoS Biol. 2018 Jan 25;16(1):e2003992. doi: 10.1371/journal.pbio.2003992 (PMC5784887; doi:10.1371/journal.pbio.2003992)

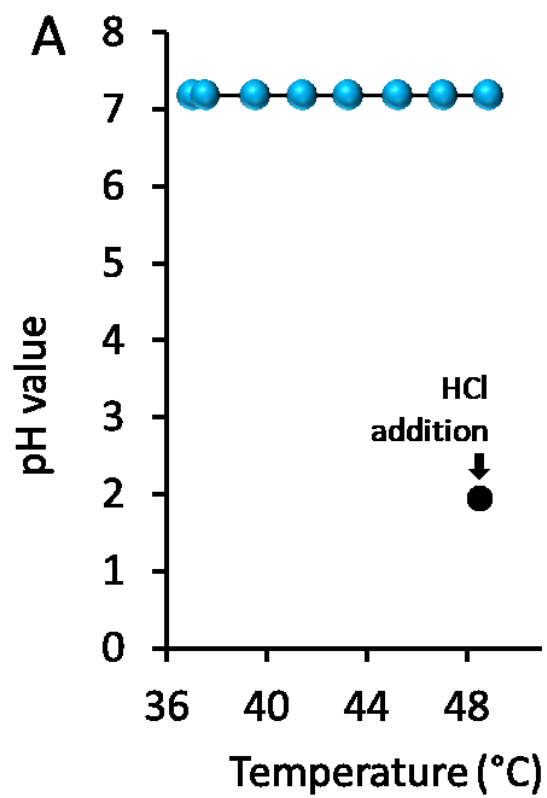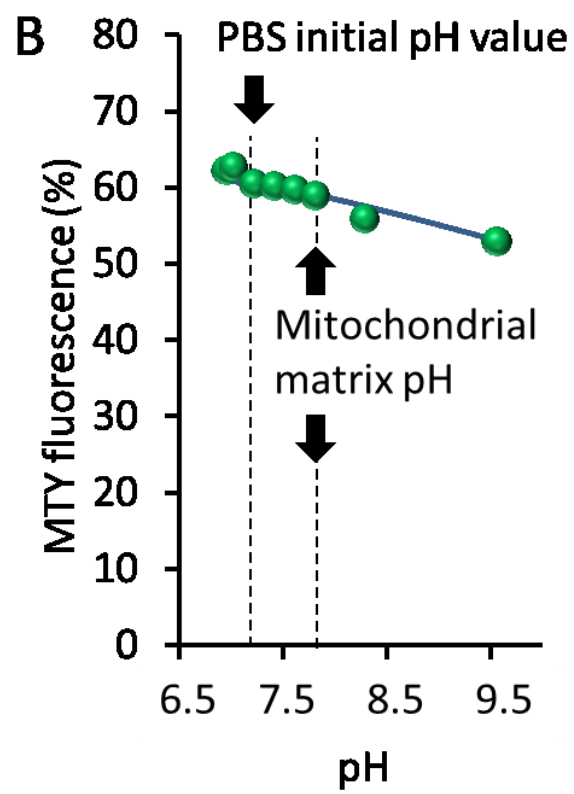

Supplement: S2 Fig — (A) Temperature did not detectably affect the pH value of PBS in the range of temperature studied (36–50 °C). (B) Change of PBS pH from 6.8 to 9.5 has only a minimal effect (<3%) on the fluorescence of MTY (1 mM). Approximately a 1% change in fluorescence was recorded as pH was raised from 7.2 to 8.0 (physiological range). Of note, a pH value of 7.8 for the mitochondrial matrix has been determined in human ECV304 (ECACC 92091712) cells [31]. Graphic drawings, means, and standard deviations are from values accessible in S2 Data. MTY, MitoThermo Yellow. (PDF) [file pbio.2003992.s002.pdf]

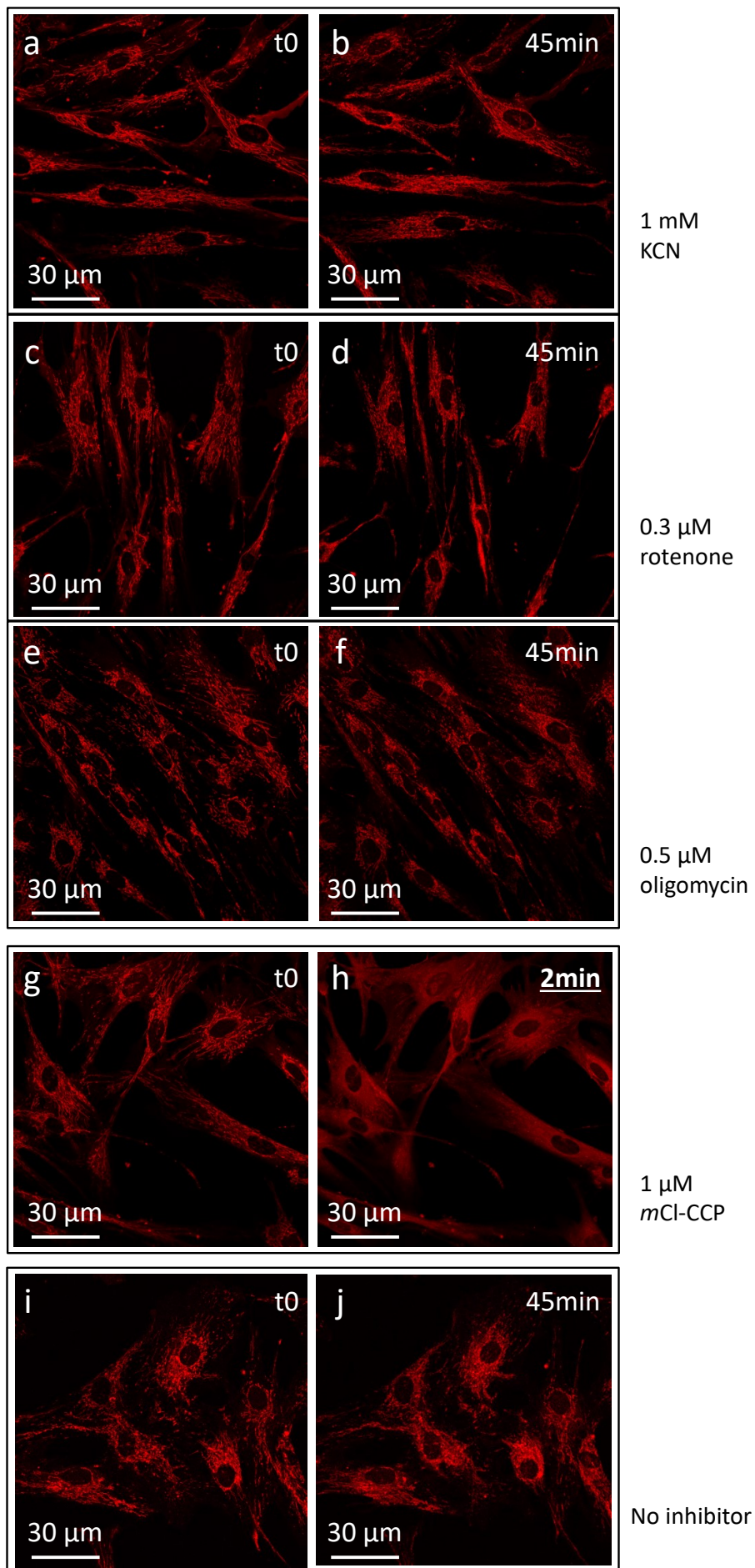

Supplement: S3 Fig — (a, c, e, g, i) At t0, before the addition of the drugs, the mitochondrial network of plated primary skin fibroblasts is clearly visible when stained with the MTY probe. After 45 min, no significant change in staining was observed when no inhibitor was present (j) or when KCN (1 mM) (b), rotenone (0.3 μM) (d), or oligomycin (0.5 μM) (f) were added. In contrast, the addition of m-Cl-CCP (1 μM) (h) rapidly causes leakage of the probe from the mitochondria, and after just 2 min, MTY appears mostly as a diffuse staining of the cytosol. Noticeably, in the presence of m-Cl-CCP (or valinomycin) treatment, anaerobiosis was unable to trigger any changes in MTY fluorescence monitored, as in Fig 1C. m-Cl-CCP, carbonyl cyanide m-chlorophenylhydrazone; MTY, MitoThermo Yellow. (PDF) [file pbio.2003992.s003.pdf]

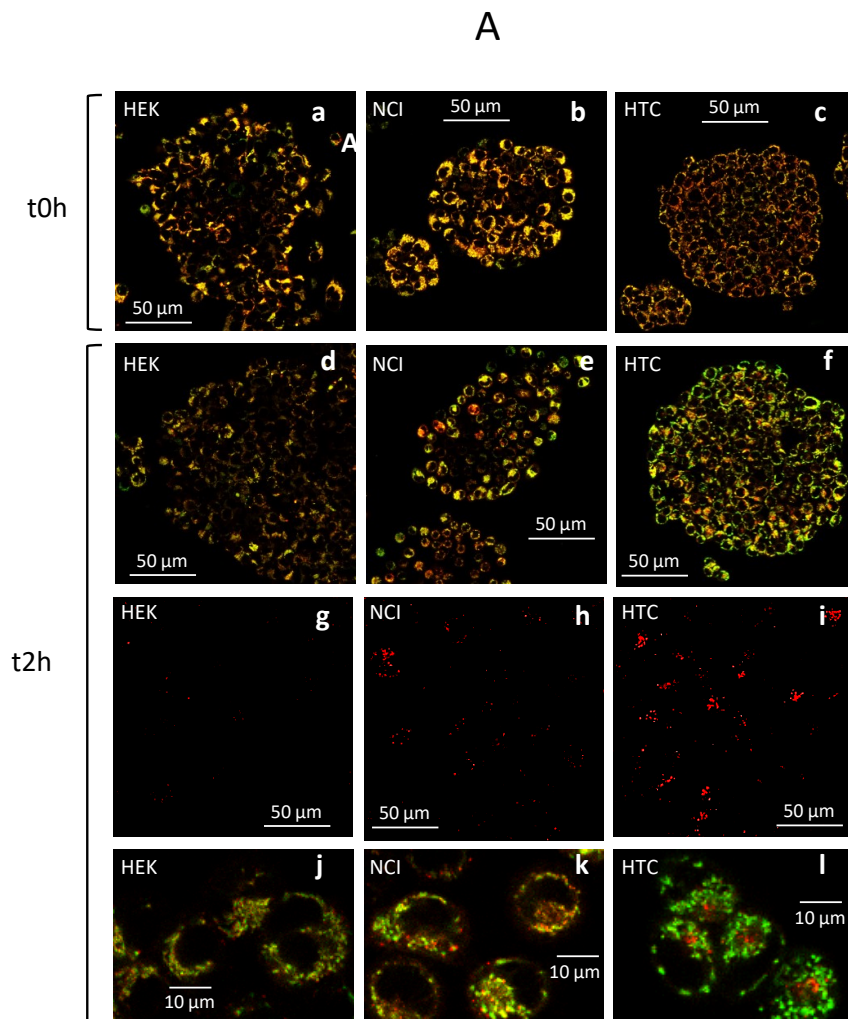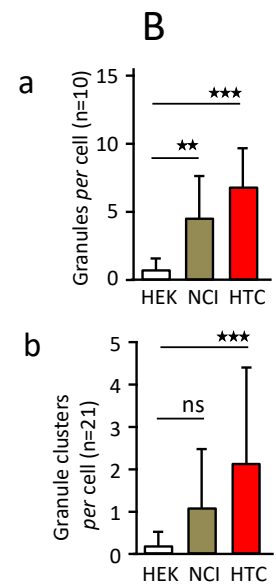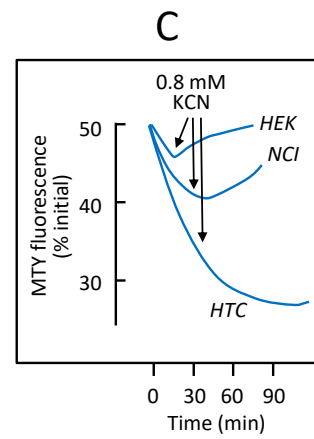

Supplement: S4 Fig — (A) Initially, MTY fluorescence is mostly localized to mitochondria in HEK293 (a), large cell lung cancer-derived cells (NCI-H460) (b), and colorectal carcinoma-derived cell line (HTC-116) (c) cell lines, as shown by the overlapping staining of MTY (shown in red) and MitoTracker green (overlapping shown in yellow). After 2 h, a significant amount of the probe is excluded from mitochondria in NCI cells (e), resulting in many cells in which green- and red-colored fluorescence is no longer fully colocalized. Notably, red (MTY) fluorescence is observed in small cytosolic granules (h). A similar but more pronounced phenomenon is observed in HTC cells, in which large granules can be observed (f, i, j). (B) Quantification of MTY-stained (red) granules (a) and clustered granules (b) in HEK, NCI, and HTC cells. (C) MTY-fluorescence changes (as in Fig 2A) in HEK, NCI, and HTC cells upon shifting from anaerobic to aerobic conditions and the effect of a subsequent addition of cyanide. Note that, while cyanide restores the initial fluorescence value in HEK cells, it does not do so in NCI and even less in HTC cells. Taken together, these experiments indicated that, depending on cell type, MTY can either be retained for at least 2 h in mitochondria (HEK cells) or excluded with variable kinetics as cytosolic granules (NCI, HTC cells), with an irreversible loss of MTY fluorescence, as measured in the spectrofluorometer quartz cuvette (C). Graphic drawings, means, and standard deviations are from values accessible in S2 Data. HEK, human embryonic kidney; MTY, MitoThermo Yellow. (PDF) [file pbio.2003992.s004.pdf]

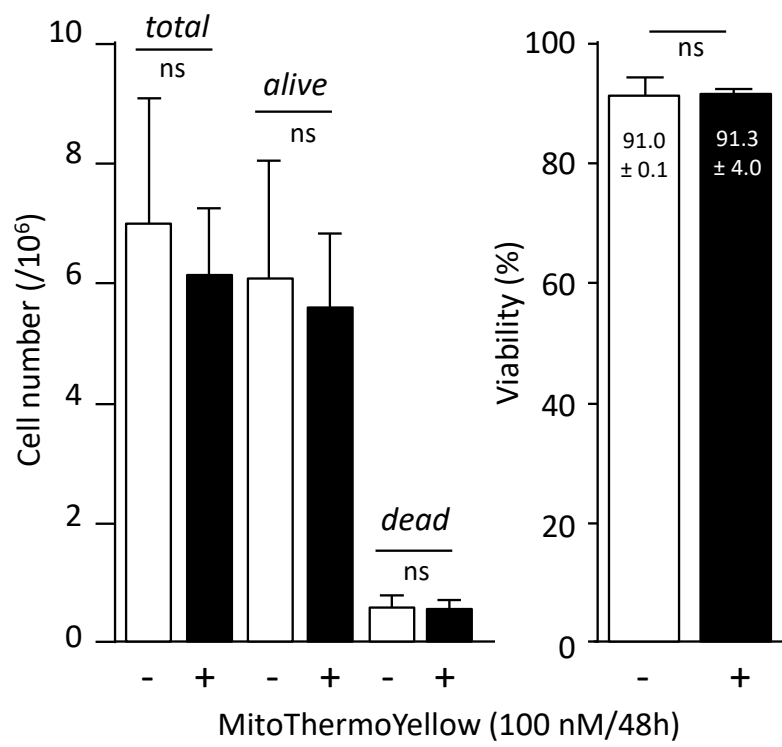

Supplement: S5 Fig — Cell counts were similar for living or dead HEK cells in the absence (−) or presence (+) of 100 nM MTY at 48 h. As a result, cell viability does not appear to be affected by MTY at this concentration. Graphic drawings, means, and standard deviations are from values accessible in S2 Data. HEK, human embryonic kidney; MTY, MitoThermo Yellow. (PDF) [file pbio.2003992.s005.pdf]

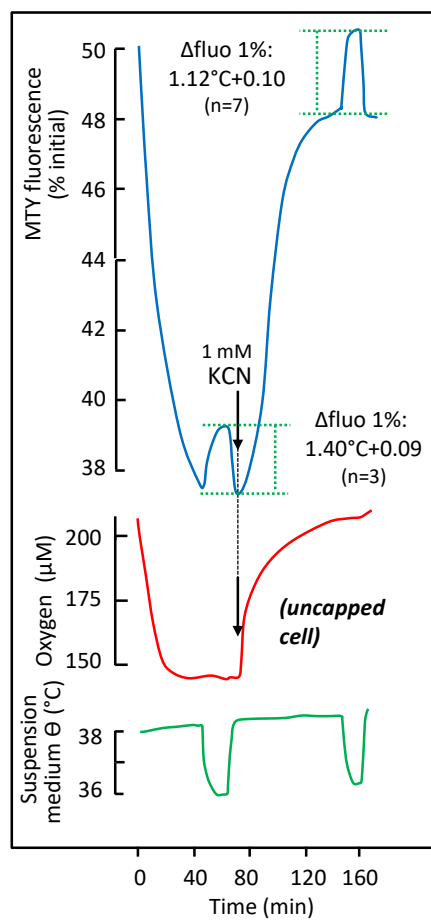

Supplement: S6 Fig — As shown in Fig 1Bb, the fluorescence response of MTY probe to temperature change tends to be decreased at high temperature compared to the response at 38 °C. At 50 °C (maximal decrease of MTY fluorescence), similarly to the probe in solution, a 2 °C shift results in 80% of the response observed at 38 °C. At such a high temperature, a 1% fluorescence change represents 1.4 °C, compared to 1.12 °C at 38 °C. HEK, human embryonic kidney; MTY, MitoThermo Yellow. (PDF) [file pbio.2003992.s006.pdf]

A

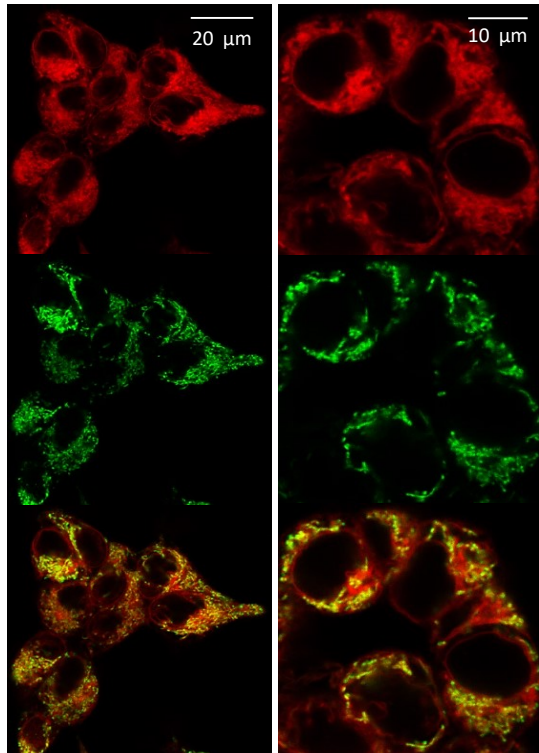

B

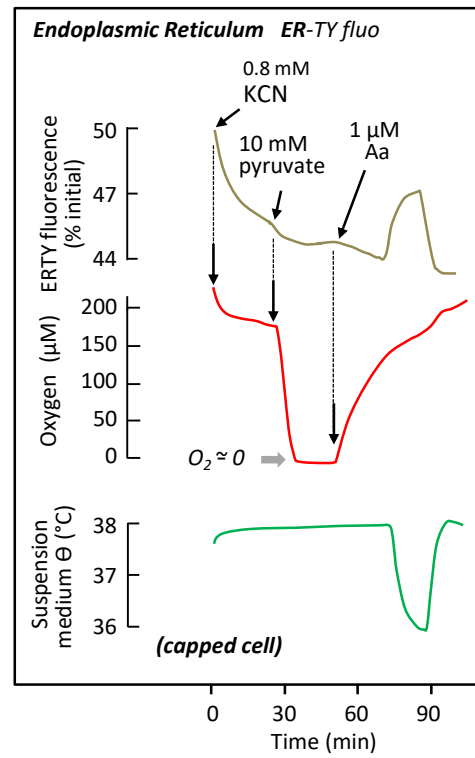

C

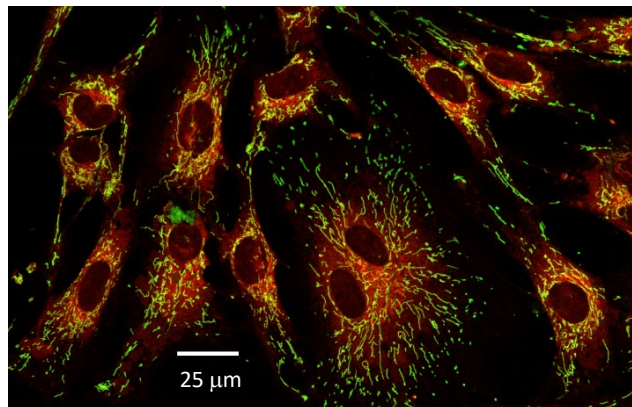

Supplement: S7 Fig — (A) ERTY (red) and MitoTracker green (green) fluorescence did not overlap (bottom) in HEK cells. (B) Tested in the same way as MTY in the spectrofluorometer (Fig 1), the fluorescence of ERTY within HEK293 cells (brown line) was unaffected by the activity of the mitochondria when modulated by cyanide, pyruvate, or antimycin, chemicals that influenced oxygen uptake (red line). Notably, the initial fluorescence decrease of ERTY was similar in the absence or presence of cyanide (not shown). (C) ERTY (red) and MitoTracker green (green) fluorescence also did not overlap in skin fibroblasts (C), in contrast to MTY and MitoTracker green, the staining of which was perfectly overlapping in the same cells (Fig 1Af). ERTY, ER Thermo Yellow; HEK, human embryonic kidney; MTY, MitoThermo Yellow. (PDF) [file pbio.2003992.s007.pdf]

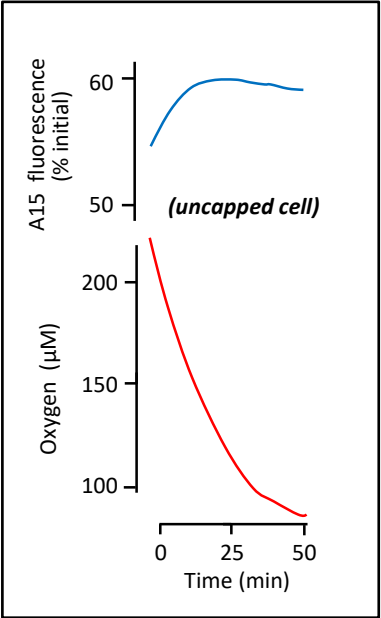

Supplement: S8 Fig — The temperature response of the MTY-related dye A15 (structure shown in S1 Fig) inside cells was previously shown to be only approximately 10% that of the temperature response of MTY itself [1]. Thus, it provides a useful control as to whether the drop in fluorescence of MTY observed in response to the activation of mitochondrial respiration is due to the previously demonstrated temperature-responsiveness of MTY, or to some other property of this family of dyes. As shown here, when cells were loaded with A15 instead of MTY and respiration was activated by oxygenation of the medium, the fluorescence changes were very different from those observed with MTY itself. Whereas MTY fluorescence showed a reversible decrease in response to respiratory activation (Fig 1C), A15 fluorescence initially showed an increase, subsequently stabilizing and drifting slightly downwards as oxygen consumption declined. Of note, the photomultiplier tension determined by the fluorimeter to result in an initial 50% fluorescent signal (condition of Fig 1C) was 650 mV with A15 (about 500 mM for MTY). This difference renders hazardous a quantitative comparison between the recorded signals with the two probes. Nevertheless, the opposite behavior of A15 is consistent with the fluorescence changes of MTY, reflecting the specific properties of the latter as a temperature sensor. HEK, human embryonic kidney; MTY, MitoThermo Yellow. (PDF) [file pbio.2003992.s008.pdf]
