# Supplementary material for: Mitochondria are physiologically maintained at close to 50 °C
Source: PLoS Biol. 2018 Jan 25;16(1):e2003992. doi: 10.1371/journal.pbio.2003992 (PMC5784887; doi:10.1371/journal.pbio.2003992)
Supplement: S1 Text — (DOCX) [file pbio.2003992.s011.docx]

**S1 Text**

Here we present calculations and approximations to assess how far our inference of a 10°C increase above ambient temperature of the probe-containing compartment of the mitochondria is compatible with the energy actually released by the mitochondria.

Based on a measured respiration of 11 nmol O2/min/mg protein, it is possible to compute the amount of NADH consumed, as 22 nmol/min/mg protein. This is because 2 molecules of NADH, supplying 4 electrons, are necessary to reduce one molecule of oxygen to water (this assumes that all of the consumed oxygen is ultimately reduced by NADH). Coupled to this NADH oxidation, up to 66 nmol ADP/min/mg protein could be phosphorylated to ATP (ADP/O=3). However, respiration in vivo does not take place under state 3 conditions (with ADP freely available) but rather in an intermediate condition between state 4 (no ADP available) and state 3, i.e. ADP/O ≈ 1.5. This corresponds to 33 nmol ATP produced per min and mg protein. The energy needed to produced 1 mol of ATP is 30.5 kJ or 7.3 kcal (i.e. 7.3 μcal/nmol). The energy released by oxidation of 1 mol of NADH to NAD^+^ is 220 kJ or 52.6 kcal/mol, or 52.6 μcal/nmol. The 22 nmol NADH/min/mg protein used to consume the oxygen therefore releases 1.157 mcal/min/mg protein (52.6 μcal x 22) or for 20 min, 23.144 mcal (1.157 x 20). Note that, throughout our study, 1 mg of cell protein was consistently used in the experiments (except where indicated; Fig 1 e,f).

If 33 nmol of ATP are produced per min and mg prot concomitantly with respiration (11 nmol O2/min/mg prot), 241 μcal/min/mg (7.3 x 33) or 0.241 mcal/min/mg are conserved as ATP, while during the same time NADH oxidation releases 1.157 mcal/min/mg. This implies that the remaining 0.916 mcal/min/mg prot are released as heat (18.32 mcal for 20 min). If one considers an ADP/O=3 (fully state 3 conditions), then this figure would be decreased to 0.675 mcal/min/mg prot (13.5 mcal for 20 min). In both cases, most of the released energy is not used for ATP production. At most 3 of the hypothetical 7 ATP molecules generated by NADH oxidation (52.6/7.3=7.2) appear to be produced.

The energy produced as heat by the respiratory chain is initially released into the inner membrane of mitochondria. In a liver cell rich in mitochondria these represent about 20% of cell volume (or weight, if considering mitochondria as having the density of water) [1] In our case (1 mg cell protein used), mitochondria would be less than 0.2 mg. If we consider the possibility that the probe is distributed throughout the entire mitochondrial matrix, representing 50-60% of that.

In an isolated system, 1 mcal can bring about a 1°C increase of 1 mg of water. Thus, the 18.32 mcal of heat released over 20 min (NADH oxidized minus ATP formed) could increase mitochondrial temperature by 91.6 °C if mitochondria are treated as a closed system, and assuming the matrix as representing about half of the mitochondrial volume up to 183.2°C (91.6°Cx2). This would rise even further in a less mitochondrially rich cell-type, such as HEK293 cells where mitochondria volume most probably does not exceed 10% of the cell volume (366.4°C).

Our observations indicating that the temperature increase does not exceed 10-12°C over 20 min imply that up to 97% of the heat produced is conducted out of mitochondria, such that at temperature equilibrium (~50°C) all of the released heat is being conducted away (Fig. 1d, phase II). This huge loss and the resulting reduced imbalance (10-12°C) of temperature (although the MTY containing compartment is sandwiched between warming membranes) presumably originate from the quite limited thermic insulation of the tiny mitochondria from cell suspension medium. Noticeably in intact cells, three membranes (plasmalemma, outer and inner mitochondrial membranes, with quite different lipid/protein composition) successively separate cell suspension medium, cytosol, and mitochondrial intermembrane space, from the probe-containing mitochondrial matrix with six (2 per membrane) water/non polar lipid head interfaces known to possibly provide each a limited, yet measurable, disturbance of thermal conductivity according to the lipid phase conditions [2].

1. Alberts B, Johnson AB, Lewis JD, Raff M, Roberts K, et al. (1994) Molecular Biology of the Cell. New York: Garland Publishing Inc. 1-642 p.

2. Youssefian S, Rahbar N, Lambert CR, Van Dessel S (2017) Variation of thermal conductivity of DPPC lipid bilayer membranes around the phase transition temperature. J R Soc Interface 14.
